# Traumatic Pancreatitis: A Rare Complication of Cardiopulmonary Resuscitation

**DOI:** 10.7759/cureus.1574

**Published:** 2017-08-17

**Authors:** Muhammad Aziz

**Affiliations:** 1 Internal Medicine, University of Kansas Hospital & Medical Center

**Keywords:** cardiopulmonary resuscitation, pancreatitis, cholangitis, biliary stricture

## Abstract

An elderly gentleman was successfully revived after undergoing cardiopulmonary resuscitation (CPR) for cardiac arrest. Post CPR, the patient developed acute pancreatitis which was likely complication of inappropriately delivered chest compressions which caused further complications and resulted in the death of the patient. This case underlines the importance of quality chest compressions that includes correct placement of hands by the operator giving chest compressions to avoid lethal injuries to the receiver.

## Introduction

This is an interesting rare case of an elderly gentleman who developed acute pancreatitis that was likely related to the complication of cardiopulmonary resuscitation (CPR) with the patient making a partial recovery. He developed complications related to his pancreatic injury to which he finally succumbed and passed away. This case highlights the importance of high-quality CPR as trivial mistakes can lead to fatal injuries.

## Case presentation

A 77-year-old male with past medical history of hypertension, mechanical aortic valve replacement (AVR), rectal carcinoma status post partial colectomy and colostomy was admitted with paroxysmal atrial fibrillation and volume overload. His heart was 110-120 and BP was 130/85 on initial presentation. The patient was started on diltiazem drip in the emergency department which was continued along with his home dose of warfarin. He also received 80 mg of IV Lasix given his volume overload status as evident on physical examination as the patient had positive jugulovenous distension and +1 bilateral pitting edema on lower extremities. An initial chest x-ray obtained demonstrated pulmonary vascular congestion, bilateral effusion and mild interstitial edema.

The patient underwent regadenoson stress test as part of ischemia workup which was negative. A transthoracic echocardiogram (TTE) was obtained that was significant for mildly dilated left atrium, moderate to severely impaired left ventricular systolic function with an ejection fraction estimated at 30% and normal diastolic function. No wall motion abnormality was detected on TTE. The patient was diuresed further with IV Lasix 40 mg and lisinopril and spironolactone were initiated. He underwent DC cardioversion (DCCV) as diltiazem did not convert him to sinus rhythm in 24 hours. His rhythm changed to normal sinus rhythm (NSR) post DCCV. Amiodarone was started after cardioversion for maintenance of NSR and warfarin dose was adjusted.

Following the night after DCCV, the patient underwent cardiac arrest and CPR was initiated. CPR continued for 60 minutes with the patient being in torsades de pointes (ventricular fibrillation) for the first 50 minutes after which the rhythm changed to pulseless electrical activity. Return of spontaneous circulation (ROSC) was achieved after 60 minutes. The patient was immediately intubated and shifted to cardiac intensive care unit. Blood pressure was initially maintained with four pressors (epinephrine, norepinephrine, dopamine, vasopressin) with a target mean arterial pressure (MAP) of greater than 65. Continuous renal replacement therapy (CRRT) was begun for acute renal failure with anuria complicated with severe anion gap metabolic acidosis. The patient also developed lactic acidosis, leukocytosis and shock liver as evident with elevated liver function tests (LFT) and International Normalized Ratio (INR). He was also started on piperacillin/tazobactam for suspected infection and pan-cultures were obtained. Pain was controlled with fentanyl drip.

**Figure 1:**
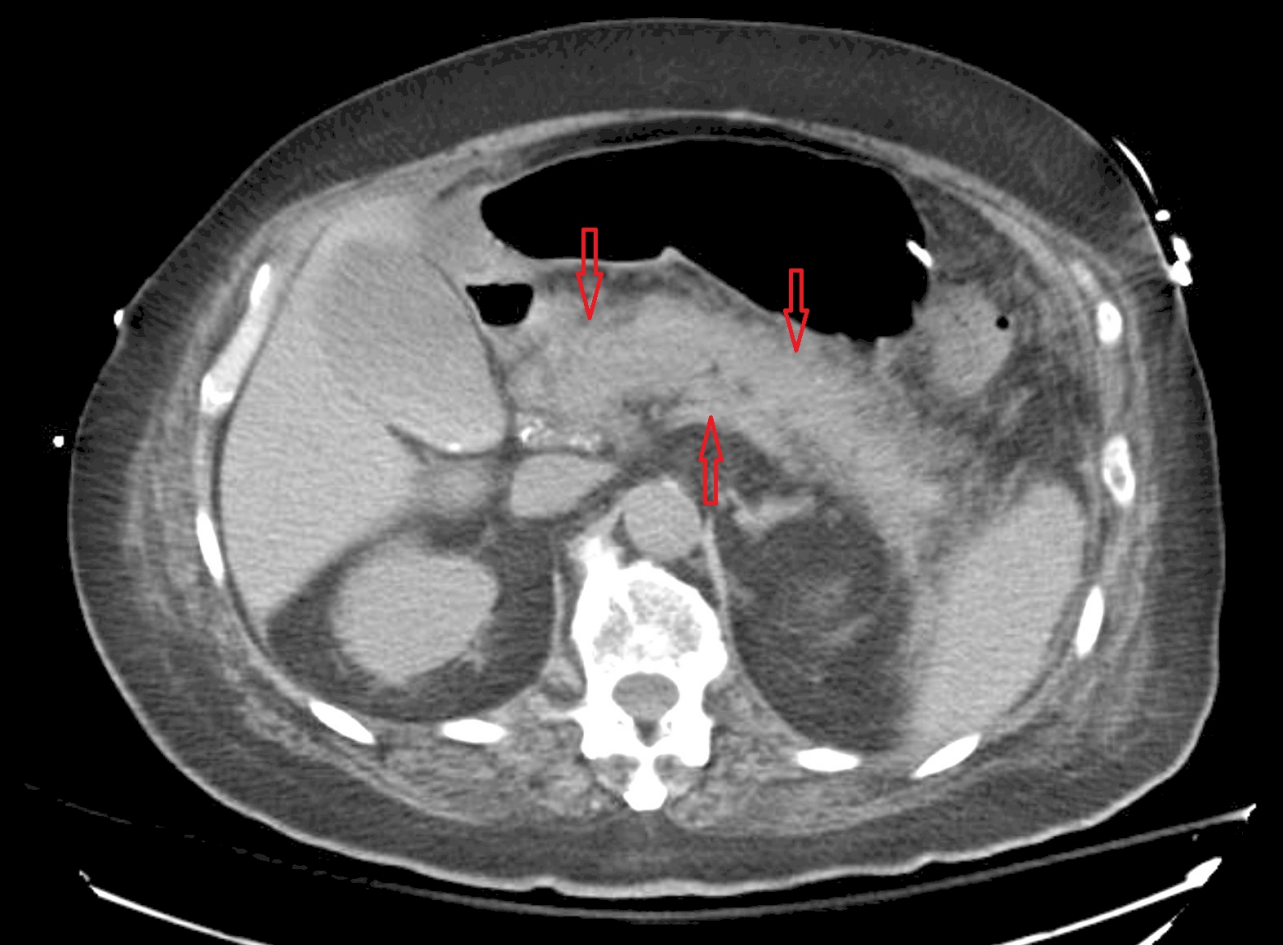
Acute pancreatitis. Computed tomography (CT) of abdomen demonstrating diffusely enlarged pancreas with indistinct margin due to inflammation representing acute pancreatitis (red arrows pointing towards inflamed pancreas).

**Figure 2:**
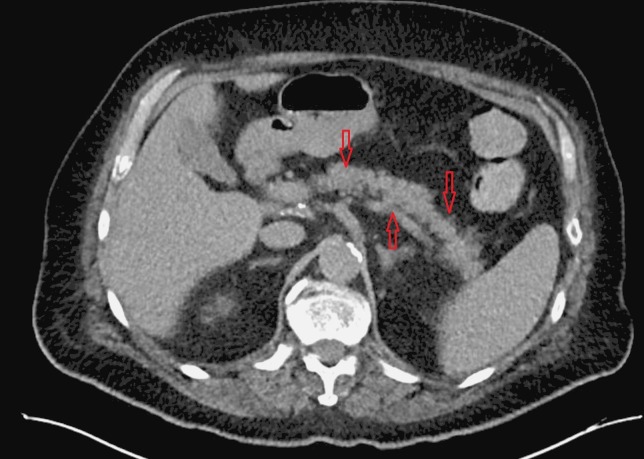
Normal pancreas. CT-angiography (CTA) of abdomen demonstrating normal pancreas (red arrows). Note the distinct border without any enlargement.

Over the course of next few days, the patient's blood pressure improved and he was weaned off all vasopressors. He became more awake and alert and responded to commands. Tracheostomy was performed for prolonged mechanical ventilation. The patient had worsening point of care (POC) blood glucose and elevated ketones in the blood and urine. He was started on insulin drip which resulted in better control of his POC glucose. Antibiotics were switched to meropenem and linezolid for worsening leukocytosis and micafungin was added for fungal coverage. Abdominal computed tomography (CT) was obtained which demonstrated acute pancreatitis (Figure 1). Lipase was sent that came back normal. A CT-angiography (CTA) obtained prior to cardiac arrest as part of the workup for abdominal aortic aneurysm was unremarkable for any signs of pancreatic abnormalities (Figure 2). Cultures obtained from the central line, peripheral, urine and sputum did not yield any organism. Lactic acidosis resolved, white blood cell (WBC) trended down and abdominal pain improved. Linezolid and micafungin were discontinued based on infectious disease recommendation as infectious etiology was less likely, however, meropenem was continued for empiric coverage for total 14 days. Insulin drip was switched to NPH insulin with correction factor while the patient was on tube feeding. The patient was successfully weaned off of ventilator and physical therapy and occupational therapy were initiated for rehabilitation.

Unfortunately, the patient developed worsening abdominal pain and elevated bilirubin in the following days. A CT scan and ultrasound of abdomen and pelvis were consistent with the development of cholecystitis without any evidence of gallstones. The patient underwent endoscopic ultrasound (EUS) with endoscopic retrograde cholangiopancreatography (ERCP) which revealed 2-3 cm stricture in the common bile duct. Pancreatic duct and common bile duct were stented and good bile flow was demonstrated. Despite this, the patient continued to endorse abdominal pain and enteral feeding was paused. Total parenteral nutrition (TPN) was initiated to provide bowel rest. The patient developed septic shock with elevated lactate and BP was barely managed with four pressors. Family was counseled and the patient passed away shortly after he was transitioned to comfort care.

## Discussion

Acute pancreatitis has multiple etiologies listed in the literature with gall stones and ethanol being the most common culprit [1]. Our patient did not have gall stones on the CT scans obtained before and after cardiac arrest. Triglyceride was found to be normal on lipid profile obtained as part of cardiac workup. Hypercalcemia was also ruled out as the patient’s calcium was low to normal throughout the hospital stay. The patient did not receive any drug notorious for causing pancreatitis including steroids, DPP4 inhibitors, protease inhibitors or nucleotide/nucleoside reverse transcriptase inhibitors during or in the month prior to hospital admission.

It was noted during the CPR that chest compressions were being wrongly delivered over the Xiphoid process of sternum for the first few minutes which was likely due to the patient’s broad stature. This may have likely caused trauma over the underlying structures including the pancreas. The chest compression delivery point was repositioned by the code leader and good quality chest compressions were ensured throughout the duration of CPR. We propose that interplay of trauma and ischemia likely caused an insult to pancreas leading to inflammation. Incidentally, the patient had CTA abdomen performed for evaluating aortic aneurysm 1 day prior to cardiac arrest and it did not reveal pancreatitis at that time (Figure 2). A subsequent CT abdomen was definitely conclusive of acute pancreatitis given the abdominal pain and tenderness following CPR.

Pancreatic injury has been described previously in the literature following CPR. In one instance the patient was resuscitated using LUCAS™ Chest Compression System, however, the patient suffered further complications including pancreatic pseudocyst, pancreatic fistula and ultimately hemorrhagic pancreas leading to death [2]. In another case, a 58-year-old gentleman suffered pancreatitis following cardiac arrest for 80 minutes during which he got continuous CPR but ultimately the patient passed away [3]. There has been one other occurrence where an 8-year-old child received intra-abdominal compressions as part of CPR protocol in pediatric population. CPR lasted >30 minutes and was unsuccessful and hemorrhagic pancreas was discovered on final autopsy [4].

As previously described in the literature, our patient suffered pancreatitis following CPR. To the best of our knowledge, this is the first instance in literature where the patient survived for almost two months despite suffering from pancreatitis following CPR possibly due to early recognition of incorrect compressions to the Xiphoid process. The ultimate cause of his death was septic shock secondary to cholangitis, which was likely caused by biliary stricture as a complication of acute pancreatitis. This case is clinically significant as physicians, physician assistants, nursing personnel and aides should understand the importance of quality chest compressions with correct positioning over the chest wall as inadequate and incorrect compressions can potentially result in lethal injuries.

## Conclusions

Although rare, pancreatitis can be a dreadful complication of CPR. One should exercise care while delivering chest compressions as wrongly delivered compressions can result in fatal injuries.
